# Prospective investigation of FOXP1 syndrome

**DOI:** 10.1186/s13229-017-0172-6

**Published:** 2017-10-24

**Authors:** Paige M. Siper, Silvia De Rubeis, Maria del Pilar Trelles, Allison Durkin, Daniele Di Marino, François Muratet, Yitzchak Frank, Reymundo Lozano, Evan E. Eichler, Morgan Kelly, Jennifer Beighley, Jennifer Gerdts, Arianne S. Wallace, Heather C. Mefford, Raphael A. Bernier, Alexander Kolevzon, Joseph D. Buxbaum

**Affiliations:** 1 Department of Psychiatry, Icahn School of Medicine at Mount Sinai, New York, NY USA; 2 Department of Informatics, Institute of Computational Science, Università della Svizzera Italiana, Lugano, Switzerland; 3 Department of Genetics and Genomic Sciences, Seaver Autism Center for Research and Treatment, Department of Psychiatry, Department of Pediatrics, Icahn School of Medicine at Mount Sinai, New York, NY USA; 4 Department of Genome Sciences, University of Washington, Seattle, WA USA; 5 Department of Psychiatry, University of Washington, Seattle, WA USA; 6 Department of Psychiatry, Department of Pediatrics, Friedman Brain Institute, Mindich Child Health and Development Institute, Icahn School of Medicine at Mount Sinai, New York, NY USA; 7 Department of Psychiatry, Department of Genetics and Genomic Sciences, Department of Neuroscience, Friedman Brain Institute, Mindich Child Health and Development Institute, Icahn School of Medicine at Mount Sinai, New York, NY USA; 8 Seaver Autism Center for Research and Treatment, Icahn School of Medicine at Mount Sinai, New York, USA

## Abstract

**Background:**

Haploinsufficiency of the forkhead-box protein P1 (*FOXP1*) gene leads to a neurodevelopmental disorder termed FOXP1 syndrome. Previous studies in individuals carrying *FOXP1* mutations and deletions have described the presence of autism spectrum disorder (ASD) traits, intellectual disability, language impairment, and psychiatric features. The goal of the present study was to comprehensively characterize the genetic and clinical spectrum of FOXP1 syndrome. This is the first study to prospectively examine the genotype-phenotype relationship in multiple individuals with FOXP1 syndrome, using a battery of standardized clinical assessments.

**Methods:**

Genetic and clinical data was obtained and analyzed from nine children and adolescents between the ages of 5–17 with mutations in *FOXP1*. Phenotypic characterization included gold standard ASD testing and norm-referenced measures of cognition, adaptive behavior, language, motor, and visual-motor integration skills. In addition, psychiatric, medical, neurological, and dysmorphology examinations were completed by a multidisciplinary team of clinicians. A comprehensive review of reported cases was also performed. All missense and in-frame mutations were mapped onto the three-dimensional structure of DNA-bound FOXP1.

**Results:**

We have identified nine de novo mutations, including three frameshift, one nonsense, one mutation in an essential splice site resulting in frameshift and insertion of a premature stop codon, three missense, and one in-frame deletion. Reviewing prior literature, we found seven instances of recurrent mutations and another 34 private mutations. The majority of pathogenic missense and in-frame mutations, including all four missense mutations in our cohort, lie in the DNA-binding domain. Through structural analyses, we show that the mutations perturb amino acids necessary for binding to the DNA or interfere with the domain swapping that mediates FOXP1 dimerization. Individuals with FOXP1 syndrome presented with delays in early motor and language milestones, language impairment (expressive language > receptive language), ASD symptoms, visual-motor integration deficits, and complex psychiatric presentations characterized by anxiety, obsessive-compulsive traits, attention deficits, and externalizing symptoms. Medical features included non-specific structural brain abnormalities and dysmorphic features, endocrine and gastrointestinal problems, sleep disturbances, and sinopulmonary infections.

**Conclusions:**

This study identifies novel *FOXP1* mutations associated with FOXP1 syndrome, identifies recurrent mutations, and demonstrates significant clustering of missense mutations in the DNA-binding domain. Clinical findings confirm the role *FOXP1* plays in development across multiple domains of functioning. The genetic findings can be incorporated into clinical genetics practice to improve accurate genetic diagnosis of FOXP1 syndrome and the clinical findings can inform monitoring and treatment of individuals with FOXP1 syndrome.

**Electronic supplementary material:**

The online version of this article (10.1186/s13229-017-0172-6) contains supplementary material, which is available to authorized users.

## Background

Haploinsufficiency of the forkhead-box protein P1 (*FOXP1*) gene has recently been shown to cause a neurodevelopmental disorder with a phenotype characterized by global developmental delay (DD), intellectual disability (ID), speech deficits, mild dysmorphic features, and autism spectrum disorder (ASD) traits [1–5]. Here, we refer to this disorder as FOXP1 syndrome.

Since the first report of a deletion spanning *FOXP1* and three other genes in a child with DD, speech delay, hypertonia, dysmorphic features, contractures and blepharophimosis [6], nearly 20 cases have been reported. The identification of deletions limited to *FOXP1* [1–3, 7, 8] and of several individuals with loss-of-function and missense variants in *FOXP1* [1, 3–5, 9, 10] has delineated *FOXP1* haploinsufficiency as sufficient to produce core features including delayed motor and language milestones, global speech impairment, ID, dysmorphic features, and ASD. Additional *FOXP1* mutations have also emerged from large-scale targeted sequencing or exome and genome sequencing analyses of cohorts with ID [11–13] or ASD [14, 15]; however, these studies have provided minimal phenotypic information. *FOXP1* can be among the genes deleted in cases with 3p14 deletion syndrome, a contiguous gene syndrome that also presents with hearing loss, congenital heart defects, and urogenital abnormalities [16–18]. Interestingly, common variation at the *FOXP1* locus has shown association in a cross-disorder meta-analysis of ASD and genome-wide association studies in schizophrenia [19]. Eight additional pathogenic mutations have emerged after the discovery of a de novo mutation in a whole exome sequencing (WES) study on congenital anomalies of the kidney and urinary tract [20]. Notably, all individuals have neurodevelopmental phenotypes compatible with FOXP1 syndrome and the majority (6/8) display upper or lower urinary tract defects [20].

FOXP1 is a transcription factor of the *FOX* gene family, named for the forkhead-box DNA-binding domain present in the gene family [21]. The *FOXP* subfamily is comprised of four genes: *FOXP1*, *FOXP2*, *FOXP3*, and *FOXP4*. The closest homolog to *FOXP1*, and the best-known member of the *FOXP* family, is *FOXP2*. In the brains of zebra finch songbirds and humans, Foxp1 and Foxp2 are co-expressed in the GABAergic medium spiny neurons of the striatum [22, 23], a brain region critical for human language, mouse ultrasonic vocalization (USV), and zebra finch vocal imitation. Both genes are important for language production and comprehension. In addition to the speech impairments observed in individuals with FOXP1 syndrome, maternal uniparental disomy of chromosome 7 (reducing *FOXP2* expression) [24], *FOXP2* deletions [25], and *FOXP2* mutations [26, 27], all result in childhood apraxia of speech and other speech and language defects. In addition, both *FOXP1* and *FOXP2* are critical during cortical neurogenesis and specification [28–30]. Constitutive *FOXP1* knockout is embryonically lethal due to a cardiac defect, but brain-specific conditional *FOXP1* null mice display striatal morphological defects, reduced USV (also reported in *FOXP2* mutant mice [31]) and social and cognitive deficits [23, 32].

Shared and pervasive clinical features in individuals with *FOXP1* deletions and mutations include mild-to-moderate ID, language impairment, and motor delays [1–5, 10, 13]. All reported individuals with FOXP1 syndrome displayed speech and language impairment. While delayed, the developmental trajectory of cognitive, language, and motor skills remains unknown. The majority of individuals described in the literature developed a minimum of phrase speech, all individuals were reported to have difficulty with articulation, and language was often limited to phrases or simple sentences [1, 2, 4, 5]. Several investigators reported that expressive language is more affected than receptive language [1–3]; however, these findings were not based on norm-referenced standardized testing.

Medical features of individuals with *FOXP1* mutations reported in the literature vary widely and include brain and cardiac malformations, hypotonia, strabismus, and obesity [2, 4, 5, 7, 16, 33, 34]. Dysmorphic features associated with FOXP1 syndrome appear to be mild and inconsistent. Among the most commonly reported dysmorphic features are a prominent forehead, downslanting or short palpebral fissures, and a short nose with a broad tip [3]. Other features may include widely spaced eyes, frontal hair upsweep, ptosis, and hypertelorism [7]. Behavioral anomalies, including ASD or autistic traits, aggression, anxiety, and obsessive-compulsive symptoms, were present in a majority of reported cases [1, 4, 5, 8, 10, 13].

To date, no study has prospectively evaluated more than three individuals with FOXP1 syndrome using a battery of standardized measures. The goal of the present study was to comprehensively characterize FOXP1 syndrome by utilizing a multidisciplinary team of biologists and clinicians and objective assessments to prospectively evaluate a cohort of children and adolescents with mutations in the *FOXP1* gene. We also characterize one individual with a duplication of 8.4 Mb spanning *FOXP1* and 47 additional genes, which has not previously been described and remains of unknown clinical significance (Additional file 1). Evaluating genetic results in conjunction with a robust battery of clinical assessments will better elucidate the genotype-phenotype relationship in this recently described syndrome, while functional dissection of mutations will provide insights into the pathobiological mechanisms underlying the FOXP1 syndrome.

## Methods

### Participants

Phenotypic characterization was completed in nine children and adolescents (seven female) with mutations in *FOXP1* and one female with a large duplication spanning *FOXP1* (Additional file 1). Individuals were between the ages of 5–17 (mean ± SD = 11.1 ± 3.6). Evaluations were completed at the Seaver Autism Center for Research and Treatment at the Icahn School of Medicine at Mount Sinai (*n* = 6, subjects S1-S6) and at the Center on Human Development and Disability at the University of Washington (*n* = 4, subjects W1-W4). Comprehensive medical, neurological, dysmorphology, and neuropsychological evaluations were completed by teams of child and adolescent psychiatrists, clinical psychologists, neurologists, and clinical geneticists. A battery of standardized assessments was used to examine ASD symptomatology, intellectual functioning, adaptive behavior, receptive and expressive language, fine and gross motor skills, visual-motor integration, and psychiatric features. Individual S2 was previously reported by Lozano et al. (2015) [4]. Individual S4 was previously reported by Sollis et al. (2016) (subject 1) [5]. Individual W1 was previously reported by O’Roak et al. (2011) (subject 12817.p1) [10], but without a clinical description. This study was approved by the Institutional Review Boards of both participating sites. All caregivers provided informed written consent and assent was obtained when appropriate.

### Genetic testing

All mutations were validated by Clinical Laboratory Improvement Amendments (CLIA)-certified clinical genetics testing laboratories. The mutation in S1 was identified through clinical WES by the Molecular Genetics Laboratory at the Children’s and Women’s Health Centre in Vancouver. The mutation in S2 was identified as described before [4]. The mutation in S3 was detected through clinical WES performed by Baylor Miraca Genetics Laboratories. The mutation in S4 was identified as described before [5]. The mutation in S5 was identified through clinical WES by GeneDx. Mutations in individuals S1-S5 were further validated by Sanger sequencing in the laboratory. The mutation in W1 was identified by WES as described before [10]. The mutation in W2 was identified by clinical WES performed by the Shodair Children’s Hospital. The mutation in W3 was identified through clinical WES by GeneDx. The mutation in W4 was detected by clinical WES at Victorian Clinical Genetics Services.

The Human Genome Variation Society (HGVS) guidelines for mutation nomenclature were used [35]. In all tables and figures, the cDNA and amino acid positions were annotated according to the most updated *FOXP1* RefSeq mRNA and protein sequence (NM_032682.5 and NP_116071.2). Nucleotide numbering referring to cDNA uses +1 as the A of the ATG translation initiation codon in the reference sequence, with the initiation codon as codon 1.

The following control databases were used: Exome Variant Server (EVS, http://evs.gs.washington.edu/EVS/), Exome Aggregation Consortium (ExAC, http://exac.broadinstitute.org), and genome Aggregation Database (gnomAD, http://gnomad.broadinstitute.org).

### Review of individuals with previously published *FOXP1* mutations

We searched the published literature for mutations in *FOXP1* using PubMed, the Human Gene Mutation Database (HGMD) Professional (Biobase), and denovo-db [36]. We retrieved and examined the genetic information from all studies indicated in Additional file 2: Table S1. We also included pathogenic mutations reported in ClinVar (NCBI, http://www.ncbi.nlm.nih.gov/clinvar/). All mutations were annotated on NM_032682.5 and NP_116071.2.

### Validation of the splice site mutation in individual S1

Peripheral blood samples from individual S1 and her parents were collected in PAXgene® RNA tubes (Qiagen) and total RNA was extracted and purified using the PAXgene® Blood RNA kit, v2 (PreAnalytix). Globin mRNA was depleted from the samples using the GLOBINclear-Human Kit (Life Technologies). The quantity and quality of the purified RNA samples were measured on a Nanodrop. Total RNA (500 ng) was used for cDNA synthesis using SuperScript® II reverse transcriptase (Invitrogen). cDNA was amplified using exon-specific primers in exon 6 (SDR4345: 5′-GGACAGCTCTCAGTCCACAC-3′) and exon 9 (SDR4346: 5′-AGGTGGGTCATCATGGCTTG-3′). PCR products were extracted and purified from a 1% agarose gel using the QIAquick gel extraction kit (Qiagen) and subjected to Sanger sequencing with both forward and reverse primers (Genewiz).

### Structural analyses

The average three-dimensional structure of the FOXP1 DNA binding domain was extracted from PDB #2KIU, which contains 20 NMR structures [37], and then superimposed to the FOXP2 domain co-crystallized with one double-stranded DNA molecule (PDB #2AO7) [38]. We mapped all missense and in-frame mutations from this and prior studies on to this structure. Visual inspection of the FOXP1 domain structure and the three-dimensional superposition was performed using UCSF Chimera 1.10.1 [39]. Figures were prepared using UCSF ChimeraX.

### Clinical evaluation

A detailed clinical evaluation was completed, including medical history, psychiatric and neurological evaluation, and dysmorphology examination by clinical geneticists. Medical records were also reviewed, including magnetic resonance imaging (MRI) and electroencephalogram (EEG).

#### ASD symptomatology

ASD diagnosis was determined using the Autism Diagnostic Observation Schedule, Second Edition (ADOS-2) [40], the Autism Diagnostic Interview-Revised (ADI-R) [41, 42], and a clinical evaluation with a child and adolescent psychiatrist or clinical psychologist. The ADOS-2 is a semi-structured direct assessment of social communication and restricted and repetitive behavior. The ADOS-2 produces a total score and domain scores in the areas of Social Affect and Restricted and Repetitive Behavior. Two clinically meaningful cut-offs can be obtained: *autism spectrum* and *autism,* the latter reflecting a greater number of symptoms. Symptom severity was assessed for both total score and individual domain scores [43] using comparison scores ranging from 1 to 10. The ADI-R is a structured caregiver interview that assesses ASD symptoms in the areas of socialization, communication, and restricted and repetitive behavior. Caregiver questionnaires were completed to further assess everyday functioning and included the Social Responsiveness Scale, Second Edition [44], the Repetitive Behavior Scale-Revised [45, 46], and the Short Sensory Profile [47]. A consensus diagnosis was determined for each individual based on ADOS-2, ADI-R, and clinical evaluation (DSM-5).

#### Intellectual and adaptive functioning

Global cognitive ability was measured using the Stanford Binet Intelligence Scales, Fifth Edition (*n* = 5) [48] and the Differential Ability Scales – Second Edition (*n* = 3) [49]. Records from a Wechsler Preschool and Primary Scale of Intelligence – Third Edition [50] was reviewed for one participant (W4). Full scale IQ (FSIQ), nonverbal IQ (NVIQ), and verbal IQ (VIQ) were obtained for 7/9 participants and ratio IQs were calculated for 2/9 participants (W1, W3). Adaptive behavior was measured using the Vineland Adaptive Behavior Scales, Second Edition, Survey Interview Form [51]. The presence of ID was based on results from cognitive testing and the Vineland-II.

#### Expressive and receptive language

Language milestones were assessed during the ADI-R and the clinical evaluation. Current expressive and receptive language abilities were measured using the Vineland-II (*n* = 9), the Peabody Picture Vocabulary Test, 4th Edition [52] (*n* = 8), and the Expressive Vocabulary Test, 2nd Edition [53] (*n* = 7).

#### Gross motor, fine motor, and visual-motor integration

Motor milestones were assessed during the ADI-R and the clinical evaluation. Current fine and gross motor skills were measured using the Vineland-II (*n* = 5). Visual-motor integration was measured using the Developmental Test of Visual-Motor Integration, Sixth Edition [54] (*n* = 7), which required individuals to copy shapes and patterns using pencil and paper. The Developmental Coordination Disorder Questionnaire [55] was completed by all caregivers to measure motor control during movement, fine motor/handwriting skills, and general coordination. Scores below 57 are indicative of a developmental coordination disorder.

#### Psychiatric features

The presence of internalizing and externalizing psychiatric symptoms was assessed during the psychiatric evaluation and through caregiver report forms including the Achenbach Child Behavior Checklist [56] and the Aberrant Behavior Checklist [57–59].

## Results

### *FOXP1* mutational spectrum

**Fig. 1:**
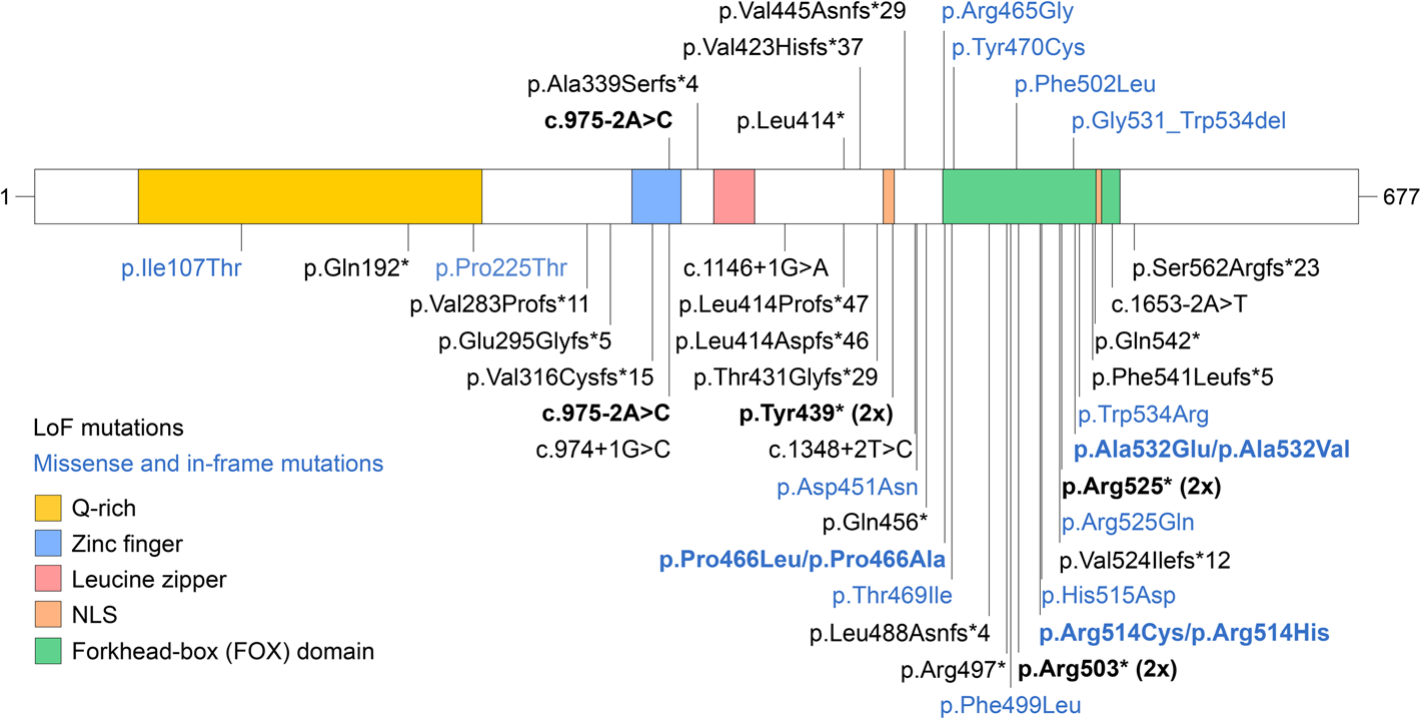
*FOXP1* mutations. The mutations described in this study and those described in the literature are shown in the upper and lower panels, respectively. FOXP1 domains are reported as described for Q9H334–1 in Uniprot. The two nuclear localization signals (NLS) are indicated as previously reported [68]. Missense and in-frame mutations are indicated in blue, while loss-of-function (LoF) mutations are indicated in black. Recurrent mutations are indicated in bold. The position of c.975-2A > C reflects the Lys325Asnfs*12 mutation. The positions of the other splice-site mutations reflect the first residue of the exon downstream of the intron

**Fig. 2:**
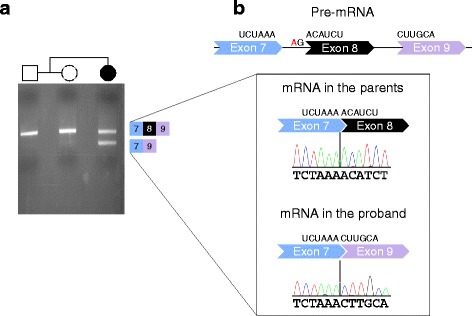
Exon skipping caused by the c.975-2A > C mutation. **a** RT-PCR results for exons 6–9 of *FOXP1* mRNA for blood-derived RNA for individual S1 and her parents. The upper band is the PCR amplicon resulting from the mRNA with exons 7 (blue), 8 (black), and 9 (purple); the lower band results from skipping of exon 8. **b** Sanger sequencing results of the PCR amplicons obtained in **a**. The nucleotide of the splice site mutated is indicated on the pre-mRNA in red

Our cohort consists of nine individuals with *FOXP1* mutations (Table 1, Fig. 1) and one individual with a large duplication encompassing the *FOXP1* gene (Additional file 1). Three individuals had frameshift mutations introducing a premature stop codon, one individual had a nonsense mutation, one individual had a mutation in an essential splice site, three had missense mutations and one had an in-frame deletion (Table 1, Fig. 1). The p.Tyr470Cys mutation is detected in a control in gnomAD and all other mutations are absent from EVS and gnomAD. Inheritance status is unknown for p.Leu414*, while all other mutations are confirmed de novo. All missense mutations are predicted to be damaging by Polyphen-2 and SIFT. Analysis of the *FOXP1* splicing in individual S1 reveals that the NM_032682.5:c.975-2A > C in the acceptor splice site between exon 7 and 8 causes exon 8 skipping and results in a Lys325Asnfs*12 mutation (Fig. 2).

**Table 1:** *FOXP1* mutations in this cohort

ID	Coding DNA change	Protein change	Genomic change	Effect	Inheritance
S1	c.975-2A>C	p.Lys325Asnfs*12	chr3:g.71050212T>G	Splice-site	De novo
S2^a^	c.1267_1268delGT	p.Val423Hisfs*37	chr3:g.71027059_71027060delAC	Frameshift	De novo
S3	c.1333_1335delinsAA	p.Val445Asnfs*29	chr3:g.71026992_71026994delinsTT	Frameshift	De novo
S4^b^	c.1393A>G	p.Arg465Gly	chr3:g.71026829T>C	Missense	De novo
S5	c.1506C>G	p.Phe502Leu	chr3:g.71026116G>C	Missense	De novo
W1^c^	c.1014dupA	p.Ala339Serfs*4	chr3:g.71050171dupT	Frameshift	De novo
W2	c.1240delC	p.Leu414*	chr3:g.71027087delG	Nonsense	Unknown
W3	c.1409A>G	p.Tyr470Cys	chr3:g.71026813T>C	Missense	De novo
W4	c.1590_1601delAGGGGCAGTATG	p.Gly531_Trp534del	chr3:g.71021757_71021768delCATACTGCCCCT	In-frame deletion	De novo

cDNA (NM_032682.5), protein (NP_116071.2/Q9H334–1), and genomic (GRCh37/hg19) changes are shown. Asterisks indicate stop codons

^a^Individual S2 was previously reported by Lozano et al. (2015) [4]

^b^Individual S4 was previously reported by Sollis et al. (2016) (subject 1) [5]

^c^Individual W1 was previously reported by O’Roak et al. (2011) (subject 12817.p1) [10], without clinical description

**Fig. 3:**
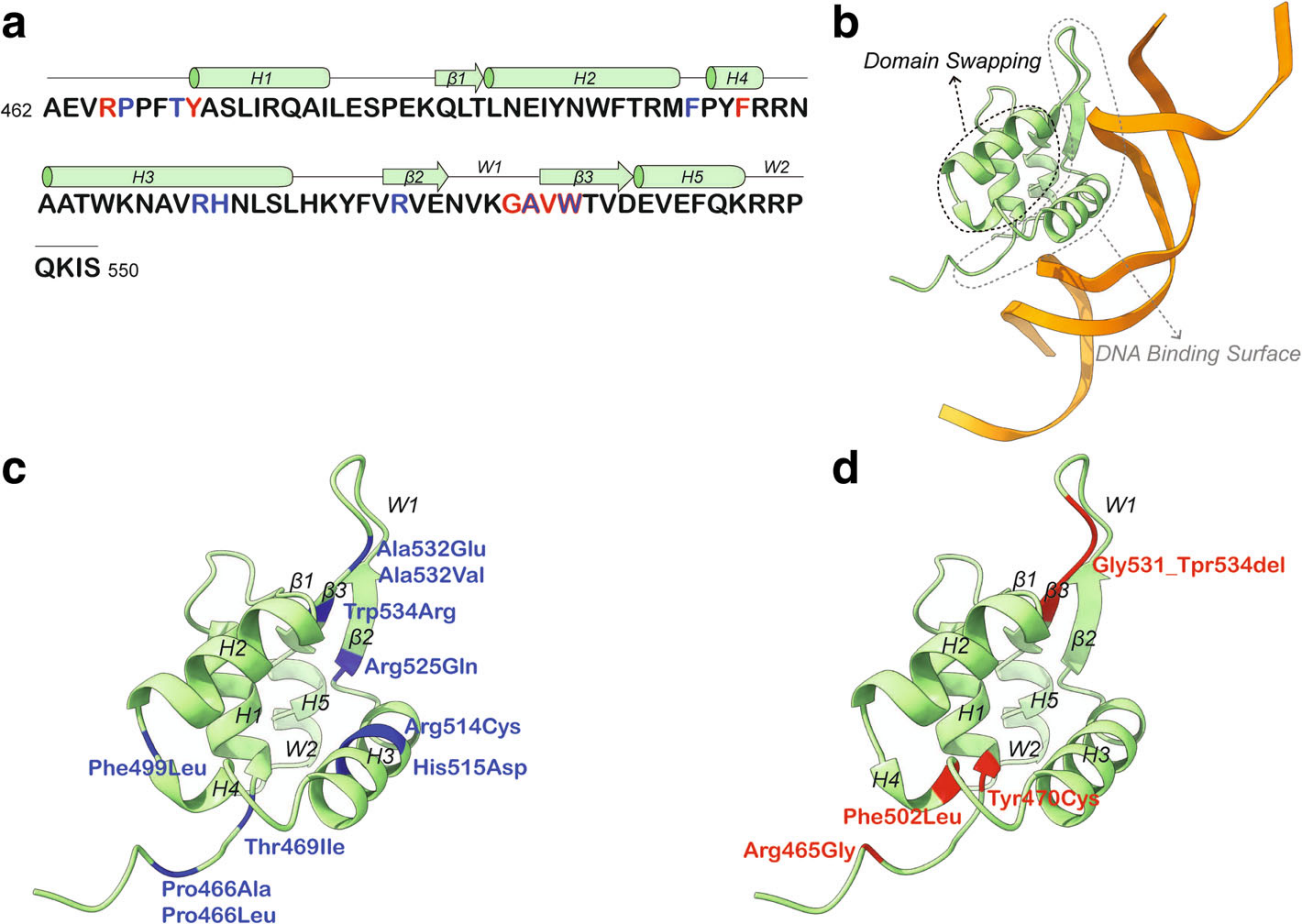
Pathogenic missense mutations in the *FOXP1* DNA-binding domain. **a** Primary sequence and topological representation of the DNA-binding domain (as reported in PDB 2KIU). The five helices (H1-H5), the three β sheet (β1-β3) and the two wings regions (W1, W2) are shown. Residues mutated in the cohort described in this study are in red, while those affected by mutations described in literature are in blue. **b** Ribbon representation of the DNA binding domain of the FOXP1 monomer interacting with one double-stranded DNA molecule. The surface for the interaction with the DNA and the region involved in domain swapping are indicated by dashed lines. **c** Ribbon representation of the unbound FOXP1 DNA binding domain showing the missense mutations reported in the literature in blue (Additional file 2: Table S1). **d** Ribbon representation of the unbound FOXP1 DNA binding domain showing the missense and in-frame mutations reported in our cohort in red (Table 1)

We searched through the published literature and ClinVar for pathogenic mutations in *FOXP1*. Besides the missense mutation with unknown inheritance reported in Worthey et al. (2013) [9], all published mutations included in Additional file 2: Table S1 are confirmed as de novo. Notably, pathogenic missense or in-frame mutations in our cohort and in previous reports cluster to the DNA binding domain of FOXP1 (Figs. 1 and 3), critical for the transcriptional activity of the protein. As shown for FOXP2, six copies of the FOX domains bind to two double-stranded segments of DNA, with two copies as monomers tightly associated with the DNA and four exhibiting domain swapping [38]. In the monomers, the ~ 100 amino acids of the winged-helix DNA binding domain are arranged in five α-helices (H1-H5) and three β strands (β1-β3) (Fig. 3a) [37, 38]. While H3, β2 and β3 are engaged in DNA recognition and binding, H2 and H4 are directly involved in the three-dimensional domain swapping (Fig. 3b).

To understand the structural impact of the *FOXP1* mutations, we superimposed the structure of FOXP1 DNA binding domain [37] on the structure of FOXP2 bound to the DNA [38] and we mapped missense and in-frame mutations from this study and previous publications. Missense mutations previously reported (Fig. 3c) and identified in this cohort (Fig. 3d) disrupt amino acids located on both the DNA binding surface and on the helices involved in dimer formation through domain swapping. The Arg465 mutated in individual S4 is directly engaged in the DNA binding and the p.Arg465Gly mutation might affect the electrostatic interaction between the N-terminal region of the domain and the negatively charged backbone of the DNA (Fig. 3b, d). Similarly, the p.Gly531_Tpr534del in individual W4 removes four residues necessary for the three-stranded antiparallel β strands, likely compromising the folding of the domain and thus affecting DNA recognition. Within this region are also two previously reported mutations: p.Ala532Val [15] and p.Trp534Arg [13] (Fig. 3c). The Tyr470 residue mutated in W3 is part of an interaction network of aromatic residues located on H1, H3, H4 and H5 (Fig. 3d) and its mutation to Cys might result in the structural destabilization of the hydrophobic core of the protein. The Phe502 residue mutated in S5 is located on H4, which is involved in the domain swapping (Fig. 3d): reduction of steric hindrance in the core of the swapped dimer resulting from the p.Phe502Leu mutation might compromise FOXP1 dimerization.

After removing cases ascertained or reported multiple times, we found seven instances of recurrent mutations (Fig. 1, Additional file 2: Table S1). The NM_032682.5:c.975-2A > G mutation resulting in the Lys325Asnfs*12 mutation is detected in an individual in our cohort (S1, Fig. 2) and in an individual from an ID cohort screened by targeted sequencing [11]. In addition, a mutation in the first nucleotide of the splice donor site (NM_032682.5:c.974 + 1G > C) was found by WES in a DD/ID cohort [12] (Fig. 1, Additional file 2: Table S1), but the consequences of this mutation on splicing have not been assessed. A p.Tyr439* mutation was reported in a Dutch individual included in Sollis et al. (2016) (individual 3) [5] and independently identified in an individual screened by the Emory Genetics Laboratory at Emory University and deposited in ClinVar (#194566) (Fig. 1, Additional file 2: Table S1). Another nonsense mutation (p.Arg503*) was found in at least two independent cases reported in ClinVar (#211038). The mutation was also reported in a large-scale WES analysis [14], but it is unclear whether this case overlaps with one of those in ClinVar. A third nonsense mutation (p.Arg525*) was reported in two independent cases [1, 12] (Fig. 1, Additional file 2: Table S1). The other recurrent mutations are all missense mutations of residues located in the DNA binding domain (Pro466, Arg514, and Ala532) (Fig. 3a). Notably, the p.Pro466Leu is equivalent to the p.Pro505Leu in *FOXP2* that has been associated with language and speech impairment [60]. Also, the p.Arg514His is equivalent to the *FOXP2* p.Arg553His mutation identified in childhood apraxia of speech [26]. Arg514 and Arg553 in FOXP1 and FOXP2, respectively, are located in the DNA binding surface that intercalates with the major groove of the DNA (Fig. 3b, c) and p.Arg553His has been shown to cause protein mislocalization, with the formation of nuclear and cytoplasmic aggregates, and abolish the binding to DNA and transactivation [61].

### Neuropsychological phenotype of FOXP1 syndrome

An extensive battery of clinical assessments was employed to delineate the core phenotype of FOXP1 syndrome. To examine the neuropsychological phenotype, gold-standard assessment of ASD symptomatology, cognitive functioning, adaptive behavior, receptive and expressive language, fine and gross motor skills, and visual-motor integration was completed for participants S1-S6 and W1-W4 (Table 2). Results for individual S6, who carries a large duplication spanning *FOXP1*, interpreted conservatively as of unknown significance (Additional file 1: Figure S1), are discussed in Additional file 1.

**Table 2:** Neuropsychological and psychiatric manifestations in individuals with FOXP1 syndrome

Neuropsychological assessments	S1	S2	S3	S4	S5	W1	W2	W3	W4	Total(%)/Average
Age (months)	71	200	133	138	96	191	134	118	117	133.1
ASD symptoms										
ASD on ADOS-2^a^	–	+	++	–	–	++	–	++	n/a	4/8 (50%)
ADOS-2 Comparison Score	3	5	7	3	3	10	1	6	n/a	4.8
ASD on ADI-R	+	+	+	–	–	+	–	–	–	4/9 (44%)
Consensus diagnosis of ASD	–	–	–	–	–	+	–	+	n/a	2/8 (25%)
Cognitive functioning (SS)										
Nonverbal IQ	95	44	56	61	73	19	59	31	70	56.4
Verbal IQ	92	44	51	59	58	15	40	24	74	50.8
Full scale IQ	93	42	51	59	64	17	52	29	68	52.8
Adaptive behavior (SS)										
Vineland-II communication	81	59	69	67	74	42	61	57	67	64.1
Vineland-II daily Living	64	47	58	58	78	38	52	62	66	58.1
Vineland-II socialization	74	54	64	69	83	42	68	55	69	64.2
Vineland-II composite	69	53	62	63	77	39	59	59	67	60.9
Language (AE in months)										
Expressive vocabulary^b^	55	78	94	95	59	n/a	81	35	n/a	71.0
Receptive vocabulary^c^	56	69	75	93	54	44	81	29	n/a	62.6
Vineland-II expressive	42	48	59	54	55	25	52	23	46	44.9
Vineland-II receptive	41	35	47	47	30	34	26	34	30	36.0
Motor skills										
Visual-motor integration (SS)^d^	78	45	<45	45	70	n/a	47	<45	n/a	< 53.6
Vineland-II gross motor (AE)	46	n/a	47	82	59	n/a	n/a	37	n/a	54.2
Vineland-II fine motor (AE)	34	n/a	69	66	68	n/a	n/a	36	n/a	54.6
Psychiatric features										
Anxiety	+	+	+	+	+	+	+	–	+	8/9 (89%)
Compulsive behaviors	+	+	+	+	+	+	+	+	+	9/9 (100%)
Attention problems	+	+	+	+	+	+	+	+	+	9/9 (100%)
Externalizing symptoms	+	+	+	+	+	+	+	+	+	9/9 (100%)

^a^ADOS-2 classification: + = autism spectrum; ++ = autism

^b^Expressive vocabulary measured by the Expressive Vocabulary Test, 2nd Edition

^c^Receptive vocabulary measured by the Peabody Picture Vocabulary Test, 4th Edition

^d^Visual-motor integration measured by the Test of Visual-Motor Integration, 6th Edition

*ADI-R* Autism Diagnostic Interview-Revised, *ADOS-2* Autism Diagnostic Observation Schedule, 2nd Edition, *AE* age equivalents, *SS* standard score, *n/a* information not available

*ASD symptoms.* Participants S1-S5 and W1-W3 received ASD diagnostic testing and a clinical evaluation to assess DSM-5 criteria for ASD (Additional file 3: Table S2). All individuals displayed ASD symptoms, however only two of the eight individuals evaluated for ASD (W1 and W3) received a diagnosis of ASD (25%) based on expert clinical consensus.

Four individuals (S1–3, W2) were administered a Module 3 of the ADOS-2 (indicating the presence of fluent speech), two individuals (S4–5) were administered a Module 2 (indicating the presence of phrase speech), and two individuals (W1, W3) received a Module 1 (indicating use of single words or no words). Four of eight individuals met criteria on the ADOS-2; three individuals (S3, W1, W3) were above the cutoff for an *autism* classification and one (S2) was above the cutoff for an *autism spectrum* classification. The two individuals who met criteria for a *clinical diagnosis* of ASD received a Module 1 of the ADOS-2, suggesting limited language ability. ADOS-2 overall comparison scores ranged from 1 to 10 (4.8 ± 2.9). Comparison scores for individual ADOS-2 domains indicated higher scores in the Restricted and Repetitive Behavior domain (6 ± 2.7) as compared to the Social Affect domain (4.8 ± 1.8*)*.

On the ADI-R (*n* = 9), four individuals were above the cutoff for ASD on all domains, six were above the cutoff on the Social domain and seven were above the cutoff on the Communication and Restricted and Repetitive Behavior domains.

ASD symptoms were further assessed through caregiver report questionnaires. Results from the Social Responsiveness Scale, 2nd Edition (*n* = 9) indicated deficits across all domains (76.4 ± 7.8). Social motivation fell within the mild range of impairment (65.2 ± 8.3) as compared to moderate impairment in social communication (74.8 ± 9.10), social awareness (74.9 ± 6.6), and repetitive behavior (75.6 ± 8.6), and severe impairment in social cognition (76.8 ± 9.5).

In the area of repetitive behavior and restricted interests, scores on the Repetitive Behavior Scale-Revised (*n* = 9) were all within one standard deviation of norms published in individuals with ASD [62]. Within this cohort, the greatest number of symptoms was reported for insistence on sameness (5.6 ± 2.7, range: 2–10). Average self-injurious behaviors scores were higher than the reference sample (4.6 ± 4.0), however scores varied across participants (range: 0–13). On the Short Sensory Profile (*n* = 5), total scores indicated definite sensory differences in three individuals, possible differences in one individual, and typical performance in one individual. An examination of individual domains indicates the greatest number of symptoms in the area of underresponsive/seeks sensation with four of five individuals (S1, S3–4) meeting criteria for a definite sensory difference in this domain.

Results from the psychiatric evaluation provided additional detail on restricted and repetitive behavior in individuals with FOXP1 syndrome. Common repetitive behaviors included intense and highly restrictive interests, stereotypic movements such as hand flapping, insistence on sameness, or adherence to routines. Caregivers also reported compulsive behaviors in all individuals, which included hoarding of small objects. Of note, five out of the nine (56%) participants were reported to engage in compulsive picking of the skin and nails.

#### Intellectual and adaptive functioning

Five individuals received the Stanford-Binet, 5th Edition (S1-S5), three individuals received the Differential Ability Scales, 2nd Edition (W1-W3), and one individual received the Wechsler Preschool and Primary Scale of Intelligence – Third Edition (W4; record review). Developmental quotients based on ratio IQs were calculated for the Differential Ability Scales as the test was administered out of the normed age range. Standard scores across the seven individuals for which deviation IQs could be calculated ranged from 42 to 93 (62.7 ± 16.5) for FSIQ, 44 to 95 (66.3 ± 16.1) for NVIQ, and 40 to 92 (61.1 ± 18.1) for VIQ. Nonverbal developmental quotients (DQ) for individuals W1 and W3 were 19 and 31, respectively, and verbal DQs were 15 and 24, respectively. Overall, results indicate a range of cognitive ability across individuals, although NVIQ and VIQ were evenly developed within individual profiles.

Results from the Vineland-II indicate that overall adaptive functioning was consistent with cognitive functioning. Standard scores on the adaptive behavior composite ranged from 39 to 77 (60.9 ± 10.7). Mean scores were comparable across domains of communication (64.1 ± 11.2), daily living skills (58.1 ± 11.6), and socialization (64.2 ± 12.2).

Overall, seven of nine individuals (78%) received a clinical diagnosis of ID, one displayed borderline intellectual functioning (S5) and one fell in the average range (S1). It is notable that S1 is the youngest participant in the sample and her cognitive functioning was significantly better developed than her adaptive functioning. This is remarkable in light of the fact that she carries an early truncating mutation (Figs. 1 and 2).

#### Expressive and receptive language

Early language milestones were delayed in eight of nine individuals. First single words emerged between 14 and 42 months (26.8 ± 11.7), and first phrases emerged between 24 and 96 months (53 ± 23.1). Current expressive and receptive language abilities were assessed using norm-referenced measures including the Expressive Vocabulary Test, 2nd Edition (*n* = 7) and Peabody Picture Vocabulary Test, 4th Edition (*n* = 8). Results indicated stronger expressive language skills (69.4 ± 17.5) as compared to receptive language skills (55.8 ± 25.9). Expressive language standard scores ranged from 35 to 86 and receptive language scores ranged from 20 to 85. An analysis of individual results indicates that expressive language standard scores were higher than receptive language scores for all participants. Language abilities were also measured through caregiver interview via the Vineland-II (*n* = 9). Overall standard scores in the Communication domain ranged from 42 to 81 (64.1 ± 11.2). Age equivalents (AE) for all language measures are presented in Table 2.

#### Gross motor, fine motor and visual-motor integration

All individuals were delayed in achieving motor milestones. First crawling (*n* = 6) was reported between 12 and 18 months (14.8 ± 2.5) and first walking unaided (*n* = 9) was reported between 18 and 33 months (22.3 ± 4.4). Standard scores on the Developmental Test of Visual-Motor Integration (*n* = 7) ranged from < 45 to 78 (53.6 ± 14.2). Five individuals scored in the impaired range (Standard score (SS) < 50) and two individuals fell in the borderline range (SS = 70 and 78). Results from the Developmental Coordination Disorder Questionnaire (*n* = 8) provided further evidence of developmental coordination difficulties in all individuals (26.3 ± 6.6, range 15–35). Difficulties were reported across all three domains. Five individuals were administered the Vineland-II motor domain. Standard scores ranged from 61 to 91 (76.6 ± 11.1) with gross motor skills (54.2 ± 17.4) and fine motor skills (54.6 ± 17.9) reported as similarly developed.

#### Psychiatric features

Results from the Aberrant Behavior Checklist (*n* = 6) indicated elevated scores on the hyperactivity/noncompliance (62 ± 3.1, range: 57–66) and irritability/agitation (60.3 ± 3.5, range: 57–66) subscales. Scores were within normal limits on the lethargy/social withdrawal, stereotypic behavior and inappropriate speech subscales. Results from the empirically based scales of the Child Behavior Checklist (*n* = 6) indicated clinically significant levels of Attention Deficit/Hyperactivity Disorder (ADHD) symptoms, oppositional defiant problems, and conduct problems (*t* scores > 70). Anxiety problems, including obsessive-compulsive symptoms, fell in the elevated range (1.5 SD above the mean).

Results from the psychiatric evaluation revealed ADHD symptoms of inattention, hyperactivity and impulsivity in all nine individuals, with different degrees of severity and impairment. Aggression (7/9, 78%), irritability (8/9, 89%), mood lability (8/9, 89%) and self-injurious behaviors (5/9, 56%) were also present in most participants causing significant impact on daily activities. All individuals presented with anxiety symptoms in some form. Sleep problems were present in four of the nine participants (44%), and were mostly characterized by difficulty initiating sleep and multiple awakenings throughout the night. Results are summarized in Table 2.

### Other medical features of the FOXP1 syndrome

In addition to the neuropsychological evaluation, a multidisciplinary team of clinicians carried out psychiatric, medical, neurological, and dysmorphology examinations.

#### Medical features

Only two individuals in our cohort were found to have a congenital heart defect and/or electrocardiogram abnormalities (Table 3). Sinopulmonary infections were common, with recurrent otitis media reported in six out of nine (67%) individuals and recurrent upper respiratory tract infection present in four out of nine (44%) individuals. Recurrent skin infections were noted in two out of nine individuals (22%). One individual (11%) had a significant history of pulmonary problems characterized by neuroendocrine hyperplasia of infancy and pulmonary hypertension; this individual required 24-h oxygen therapy for the first 3 years of life, followed by nighttime oxygen until age five. Five out of nine (56%) individuals had visual refractive errors and strabismus.

**Table 3:** Medical findings in individuals with FOXP1 syndrome

Medical feature	S1	S2	S3	S4	S5	W1	W2	W3	W4	Total (%)
Cardiac										
Congenital heart defect	–	–	–	–	+^a^	–	–	–	+^b^	2/9 (22%)
Abnormal electrocardiogram	–	n/a	n/a	n/a	+	n/a	n/a	n/a	n/a	1/2 (50%)
Sinopulmonary										
Recurrent otitis media	–	–	+	+	+	+	–	+	+	6/9 (67%)
Recurrent upper respiratory tract infections	–	–	+	–	–	+	–	+	+	4/9 (44%)
Neuroendocrine hyperplasia of infancy	+	–	–	–	–	–	–	–	–	1/9 (11%)
Pulmonary hypertension	+	–	–	–	–	–	–	–	–	1/9 (11%)
Urinary tract										
Genitourinary abnormalities	–	–	+	n/a	–	–	–	–	–	1/8 (13%)
Endocrine										
Body mass index (percentile for age)	27.8	85.8	69.8	82.1	58.7	20.2	15.4	19.7	n/a	
Overweight	–	+	–	–	–	–	–	–	n/a	1/8 (13%)
Hypothyroidism	–	–	–	–	–	+	–	–	–	1/9 (11%)
Diabetes mellitus	–	–	–	–	–	+	–	–	–	1/9 (11%)
Other medical										
Constipation	+	+	–	–	–	–	–	+	+	4/9 (44%)
Skin infections	–	–	–	+	–	–	–	–	+	2/9 (22%)
Allergies	–	+	–	–	–	+	–	–	–	2/9 (22%)
Iron deficiency	–	–	–	–	–	+	–	–	+	2/9 (22%)
Visual refractive error	–	+	+	–	+	–	+	–	+	5/9 (56%)
Strabismus	–	+	+	–	+	–	+	–	+	5/9 (56%)

^a^Pulmonary valve stenosis

^b^Patent ductus arteriosus discovered at 14 months that resolved without intervention

*n/a* information not available

Endocrine problems were fairly common in our cohort, with two out of eight (25%) individuals falling below the 3rd percentile for stature, and two out of eight (25%) above the 80th percentile for age-expected body mass index. One child had hypothyroidism and type II diabetes mellitus. Constipation affected four out of nine individuals (44%). Two out of nine individuals (22%) had iron deficiency anemia, which was associated with restless leg syndrome in one of them. One individual (S3) showed a duplicated left-sided renal collecting duct system that required surgical repair. Genitourinary abnormalities were not reported in any other cases in this cohort.

#### Neurological features

All individuals who underwent neurological examination were found to present with fine and gross motor coordination deficits and dysarthria (Table 4). Hypotonia (89%), and mild gait abnormalities (78%) were also present in the majority of individuals. Early feeding issues, such as sucking and swallowing, were present in one third of our sample. Individual S3 presented with a tethered cord diagnosed shortly after birth. All individuals had difficulty attaining bladder control, with enuresis present in three out of nine (33%) participants. Results from brain MRIs were available for seven out of the nine participants, with abnormal findings identified in six (86%) of them (Table 4) (including S4 [5] previously reported). The most common finding was enlarged ventricles (*n* = 3). In addition, we observed cases with a small partial cavum septum pellucidum (*n* = 1), mild diffuse periventricular leukomalacia (*n* = 1), and arachnoid cysts in the left hemisphere and cerebellum (*n* = 1). Two of our participants had abnormal EEG findings in the absence of clinical seizures. There was no report of signs suggestive of a seizure disorder in any individual.

**Table 4:** Neurological findings in individuals with FOXP1 syndrome

Neurological feature	S1	S2	S3	S4	S5	W1	W2	W3	W4	Total (%)
Brain imaging abnormality	+ ^a^	+ ^b^	+ ^c^	+ ^d^	n/a	–	n/a	+ ^e^	+ ^f^	6/7 (86%)
EEG abnormality	n/a	n/a	–	n/a	n/a	+	n/a	–	+	2/4 (50%)
Hypotonia	+	+	+	+	+	–	+	+	+	8/9 (89%)
Feeding issues (past/present)	+	–	–	–	+	–	–	+	–	3/9 (33%)
Dysarthria	+	+	+	+	+	+	+	+	+	9/9 (100%)
Gait abnormalities	+	+	+	+	+	+	–	+	–	7/9 (78%)
Fine/gross motor coordination deficit	+	+	+	+	+	+	+	+	+	9/9 (100%)
Hypoacusis/hearing loss	–	–	–	–	–	–	–	–	+	1/9 (11%)
Spinal cord malformation	–	–	+^g^	–	–	–	–	–	–	1/9 (11%)

^a^Mildly dilated lateral ventricles

^b^Non-enhancing subcortical and deep white matter abnormalities; incidental finding of venous angioma in left frontal lobe

^c^Prominent Virchow-Robin spaces. Small partial cavum septum pellucidum anteriorly

^d^Mild diffuse periventricular leukomalacia

^e^Arachnoid cysts (cerebellum, left hemisphere); enlarged ventricles

^f^Enlarged ventricles

^g^Tethered cord and conus medullaris terminating at the lumbar spine segment L3

*n/a* information not available

#### Dysmorphic features

**Fig. 4:**
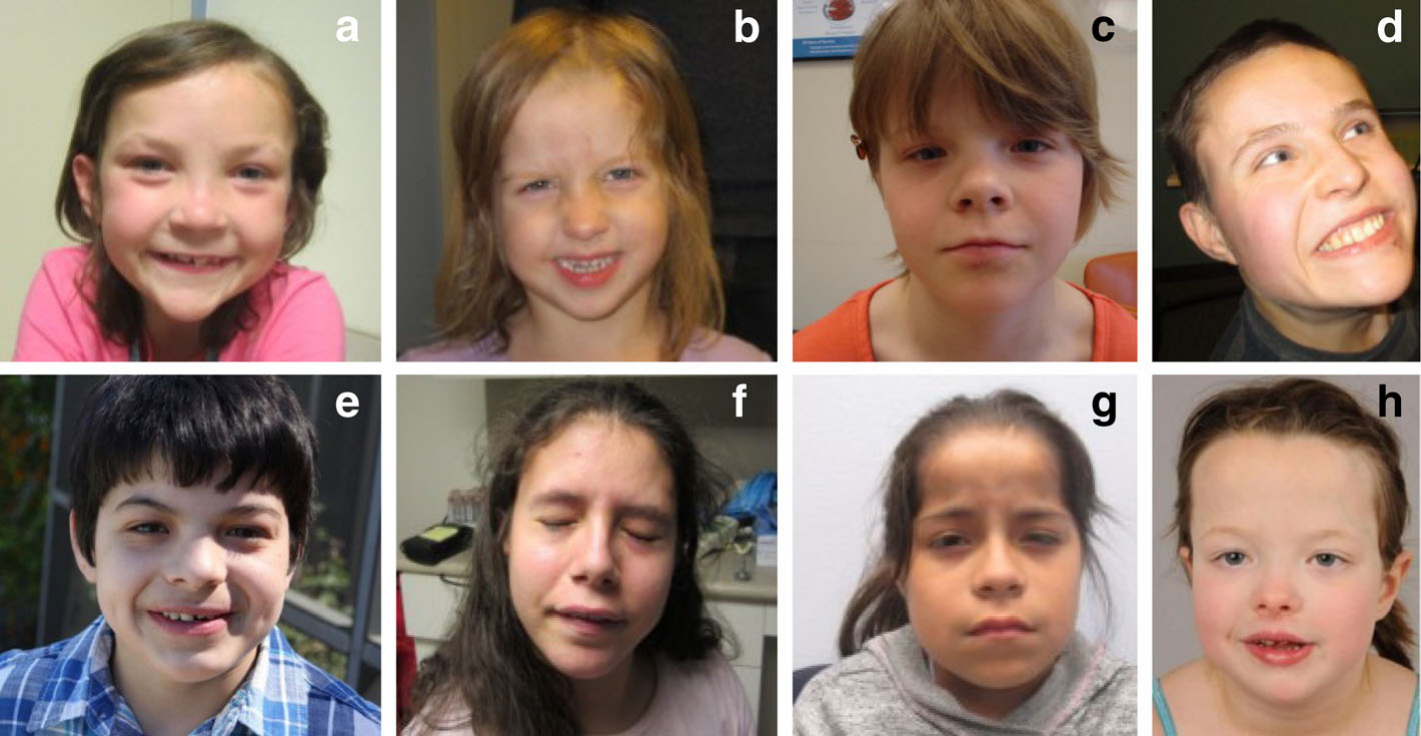
Dysmorphisms in individuals with *FOXP1* mutations. Most common features include prominent forehead (evident in **a**, **b**, **c**, **d**, **g**, and **h**), bulbous nose (evident in **a**, **c**, **e**, **f**, **g,** and **h**), broad nasal bridge (evident in **a**, **b**, **c**, **e**, and **h**), hypertelorism (evident in **a**, **c,** and **h**), thick vermillion (evident in **e**, **f,** and **h**), and long philtrum (evident in **c** and **g**)

Comprehensive dysmorphology examination was performed in eight out of nine participants (Table 5). All participants had a least three dysmorphic features (range 3–13, 7.9 ± 3.6), which is relatively consistent to previous reports. None of the identified dysmorphic features were specific to this syndrome. The most common features observed were broad nasal bridge (75%), prominent forehead (75%), bulbous nose (63%), high arched palate (50%), clinodactyly (50%), macrocephaly (50%), and hypertelorism (50%) (Fig. 4 and Table 5).

**Table 5:** Dysmorphisms in individuals with FOXP1 syndrome

Dysmorphisms	S1	S2	S3	S4	S5	W1	W2	W3	W4	Total (%)
Height in cm (percentile) Short Stature	112 (35.9) −	160.5 (35.6) −	146 (57.4) −	132.5 (2.6) +	124 (27.7) −	165.4 (24.4) −	131 (2.4) +	130 (14) −	n/a	2/8 (25%)
Macrocephaly (percentile)	−(97)	+(> 99)	−(75)	+(> 99)	+(> 99)	−(83)	−(63)	+(> 99)	n/a	4/8 (50%)
Broad nasal bridge	+	+	+	+	+	–	+	–	n/a	6/8 (75%)
Prominent forehead	+	+	–	+	+	–	+	+	n/a	6/8 (75%)
Bulbous nose	+	+	+	+	+	–	–	–	n/a	5/8 (63%)
High arched palate	+	+	+	–	+	–	–	–	n/a	4/8 (50%)
Hypertelorism	–	+	–	+	+	–	+	–	n/a	4/8 (50%)
Clinodactyly	–	–	–	+	+	+	–	+	n/a	4/8 (50%)
Epicanthal folds	–	–	–	+	–	+	+	–	n/a	3/8 (38%)
Malocclusion	+	+	–	+	–	–	–	–	n/a	3/8 (38%)
Long philtrum	–	+	+	–	+	–	–	–	n/a	3/8 (38%)
Thick vermillion	–	+	+	+	–	–	–	–	n/a	3/8 (38%)
Single palmar crease	+	–	–	–	–	+	–	–	n/a	2/8 (25%)
Pectus excavatum	+	–	+	–	–	–	–	–	n/a	2/8 (25%)
Frontal hair upsweep	+	–	–	–	–	–	–	+	n/a	2/8 (25%)
Ptosis	–	+	–	–	–	–	–	–	n/a	1/8 (13%)
Pointed chin	–	–	–	–	+	–	–	–	n/a	1/8 (13%)
Bicuspid uvula	–	–	–	–	+	–	–	–	n/a	1/8 (13%)
Partial syndactyly 2nd and 3rd toes	+	–	–	–	–	–	–	–	n/a	1/8 (13%)
Deep set eyes	+	–	–	–	–	–	–	–	n/a	1/8 (13%)
Scoliosis	–	–	–	+	–	–	–	–	n/a	1/8 (13%)
Short neck	–	–	–	+	–	–	–	–	n/a	1/8 (13%)
Sacral dimple	–	–	+	–	–	–	–	–	n/a	1/8 (13%)
Hyperflexibility	+	–	–	–	–	–	–	–	n/a	1/8 (13%)
Long eyelashes	–	–	–	+	–	–	–	–	n/a	1/8 (13%)
Total number of dysmorphic features	11	10	7	13	10	3	5	4	n/a	7.9 ± 3.6

*n/a* information not available

## Discussion

In this study, we report on the genetic and clinical spectrum of FOXP1 syndrome in a cohort of nine individuals with mutations in *FOXP1* and one individual with a large duplication spanning *FOXP1,* evaluated as of unknown significance.

Few individuals have been described in the literature, but screening the emerging *FOXP1* mutational landscape reveals 34 private mutations and another 7 that recur in unrelated individuals and appear as mutation hotspots in the gene (Fig. 1). The identification of recurrent mutations has important implications for clinical genetics practice and offers the opportunity to evaluate clinical variability in FOXP1 syndrome. For example, individual S1 has average cognitive functioning, although she carries a mutation as disruptive as other loss-of-function mutations in our cohort (Fig. 2).

Another key observation emerging from our analyses is that over 80% of the pathogenic missense mutations, including all four missense mutations in our cohort, lie in the DNA-binding domain and perturb amino acids that are necessary for the binding to the DNA or to the domain swapping that mediates FOXP1 dimerization (Fig. 3). This finding emphasizes the importance of carefully evaluating *FOXP1* missense variants and taking into account structural information when evaluating pathogenicity.

Our clinical observations delineate a clinical spectrum of FOXP1 syndrome that includes a set of core phenotypic features, including delays in early motor and language milestones, language impairment, ASD symptoms (although subthreshold for a DSM-5 diagnosis in the majority of individuals), and visual-motor integration deficits (Table 2). Psychiatric features were also prominent including anxiety, obsessive-compulsive traits, attention deficits, and externalizing symptoms. Cognitive ability ranged from profound ID to average, with the majority of individuals performing in the range of mild ID (standard scores between 50 and 70). Interestingly, there was not a significant difference between verbal and nonverbal abilities. Adaptive functioning was similarly developed, suggesting evenly developed skills.

With regard to ASD symptoms, the majority of individuals fell above ASD cutoffs on standardized diagnostic assessments (ADOS-2, ADI-R); however, only two individuals met DSM-5 criteria for ASD (Additional file 3: Table S2). It is notable that a greater number of symptoms were observed in the Restricted and Repetitive Behavior domain as compared to the Social Communication domain. While results highlight the role of clinical judgment and suggest that a high level of expertise is required to fully assess ASD, individuals with FOXP1 syndrome will nevertheless likely benefit from similar treatments to those with ASD. In addition to interventions targeting repetitive behaviors, sensory symptoms, and compulsive-like behaviors, social skills training is likely warranted given the extent of social difficulties endorsed by caregivers across measures.

In contrast to findings from previous studies [1–3], our results using norm-referenced language assessments indicate that expressive language is better developed than receptive language. Parents reported fewer skills in both expressive and receptive subdomains on the Vineland-II as compared to results from clinician-administered assessments (Expressive Vocabulary Test, 2nd Edition and Peabody Picture Vocabulary Test, 4^th^ Edition). This discrepancy likely reflects differences between language ability and the application of language during daily functioning. Visual-motor integration and motor coordination deficits were also present in all individuals. Language and motor weaknesses appear to emerge early, as evidenced by delays in the achievement of developmental milestones.

The wide age range of participants did appear to impact performance on standardized assessments, particularly cognitive testing. Within this cohort, the two youngest participants achieved the highest IQ scores and the oldest participant achieved the lowest IQ score. As individuals with developmental delays age, scores on standardized testing often declines due to an individual’s failure to gain new skills at the expected pace. Larger samples are needed to assess the progression of the syndrome and to better understand the variability in clinical presentation across patients.

The individual with the *FOXP1* duplication presented with a similar phenotype, which included delays in reaching developmental milestones, borderline cognitive and adaptive functioning, visual-motor integration deficits, sub-threshold ASD symptoms, and clinically significant levels of anxiety (Additional file 1).

On the neurological exam, fine and gross motor coordination deficits were present in all individuals, which is consistent with motor delays and continued deficits in the motor domain. Structural abnormalities on brain MRI were detected in the majority of individuals but did not follow a specific pattern. Enlarged ventricles were the most common imaging finding and this has been reported previously [3, 20]. There was no report of seizure disorder in any individual.

On the medical exam, we observed congenital heart defects in two out of six individuals examined. An association between congenital heart defects and *FOXP1* haploinsufficiency has been suggested in an earlier report [63] and sporadically detected in affected individuals [20], but not replicated in a large-scale WES study on congenital heart defects [64]. Nevertheless, *FOXP1* plays a key role in cardiac morphogenesis in mice [65] and cardiac problems should be assessed in individuals with *FOXP1* mutations. Another relevant medical finding is related to congenital anomalies of the kidney and urinary tract. A previous study reported eight individuals with de novo mutations in FOXP1, six of which had congenital anomalies of the kidney and urinary tract [20]. One individual in our cohort had congenital renal defects. While genitourinary abnormalities were not reported in the other patients in our cohort, it remains important to consider kidney/urinary tract congenital anomalies when assessing individuals with FOXP1 syndrome. In addition, constipation was reported by several parents in our cohort and is often present in children with neurodevelopmental disorders, especially in conditions associated with hypotonia. On review of the medications used in our cohort, it does not appear that the extent of constipation can be accounted for by psychotropic medication use. The majority of individuals affected by constipation were either not receiving medications, or not receiving medications where constipation is a common side effect.

On the dysmorphology exam, although non-specific, over half the cohort presented with several dysmorphic features including a broad nasal bridge, prominent forehead, bulbous nose, high arched palate, clinodactyly, strabismus, and hypertelorism (Fig. 4).

Our genetic findings can also inform the design of patient-specific cellular models and animal models with stronger construct validity, which are at this point critical to understand the underpinnings of FOXP1 syndrome. Thus far, cultured rodent neurons have been employed to identify the defects in neuronal morphology and physiology resulting from *FOXP1* silencing [66] or knockout [32]. Also, the functional consequences of *FOXP1* mutations have been investigated only in non-neuronal cells. *FOXP1* mRNA harboring loss-of-function mutations are likely to undergo non-sense mediated decay, as shown for p.Ala339Serfs4* [10], but at least a fraction of them escape non-sense mediated decay. Exogenously expressed FOXP1 mutants harboring p.Val423Hisfs*37 [4], p.Ala339Serfs4* [5, 10], p.Tyr439*, or p.Arg525* [5] have been shown to disrupt nuclear localization. Missense mutations result in aberrant aggregates in the nucleus and cytoplasm (p.Arg514Cys and p.Arg465Gly) or only in the cytoplasm (p.Trp534Arg) [5]. These eight mutations abolish the transcriptional repression activity of FOXP1, as shown by enhanced expression of a luciferase reporter [1, 4, 5]. While mutations p.Ala339Serfs4*, p.Arg525*, and p.Trp534Arg suppress the interaction with wildtype FOXP1 and FOXP2 [4, 5], FOXP1 mutants carrying p.Tyr439*, p.Arg514Cys, or p.Arg465Gly retain the ability to bind FOXP1 and FOXP1 and might exert dominant-negative effects [5]. Investigating neuronal models carrying recurrent pathogenic mutations or patient-derived human neuronal models is key to comprehensively expose the pathophysiological mechanisms underlying FOXP1 syndrome.

Similarly, mouse models studied thus far have brain-specific knockout of *FOXP1* [23, 32]. While these models have been instrumental in elucidating the fundamental role of *FOXP1* in the striatum and their relevance to some of the phenotypes observed in individuals, they recapitulate only in part the genetic architecture of FOXP1 syndrome. First, they accurately model only cases with *FOXP1* deletions rather than mutations. A strategy to mimic patient-specific mutations has been applied for *FOXP2* by generating a knockin mouse with the equivalent of the p.Arg553His human mutation (p.Arg514His in *FOXP1*) [67]. Second, these studies have used homozygous animals, while the syndrome results from a heterozygous defect, rather than complete loss of *FOXP1*. The clinical evidence reported here might inform targeted phenotypic characterization of *Foxp1* rodent models to fully capture the clinical features of the syndrome, including the neurodevelopmental phenotype and the other medical co-morbidities. Based on the clinical phenotypes in individuals with FOXP1 syndrome, a comprehensive characterization of heterozygous *FOXP1* rodent models should include an examination of motor abnormalities, hyperactivity and executive functioning, repetitive and compulsive behaviors, social and communication deficits, cognitive changes, anxiety, and circadian/sleep abnormalities. Deficits in visual-motor integration would be particularly interesting to study. In addition, careful examination of cardiac, renal and visual organs, as well as brain ventricle size would be warranted. Finally, immune and endocrine function should be investigated.

## Conclusions

This study identifies novel *FOXP1* mutations associated with FOXP1 syndrome and identifies recurrent mutations as well as a significant clustering of missense mutations in the DNA-binding domain. These findings can be incorporated into clinical genetics practice to improve accurate genetic diagnosis of FOXP1 syndrome. This study also describes a phenotype characterized by motor and language delays (expressive language better developed than receptive language), visual-motor integration deficits, ASD symptoms, and associated features of anxiety, ADHD, obsessive-compulsive traits, and externalizing behavior. Intellectual and adaptive functioning ranged from profound ID to average, with evenly developed skills within individuals. Individuals identified to have a *FOXP1* mutation should begin behavioral interventions (e.g., physical therapy, speech and language therapy, occupational therapy) as early as possible, even before delays emerge. Psychiatric interventions will be critical to appropriately manage psychiatric symptoms as they arise. In order to make informed educational recommendations, future studies should assess academic achievement in individuals with FOXP1 syndrome. Natural history studies are also critical to examine the effects of FOXP1 syndrome throughout the lifespan in order to develop improved guidelines for medical assessment, monitoring, and treatment. The careful examination of model systems will lead to improved understanding of the pathophysiology of FOXP1 syndrome and can lead to novel therapeutic targets.

## Additional files

Additional file 1: Supplementary note containing the genetic and clinical information for the individual with the *FOXP1* duplication. (DOCX 107 kb)

Additional file 2: Pathogenic *FOXP1* mutations reported in the literature and in ClinVar. (XLSX 24 kb)

Additional file 3: DSM-5 criteria for ASD in individuals with *FOXP1* mutations. (DOCX 18 kb)
