# Supplementary material for: Prospective investigation of FOXP1 syndrome
Source: Mol Autism. 2017 Oct 24;8:57. doi: 10.1186/s13229-017-0172-6 (PMC5655854; doi:10.1186/s13229-017-0172-6)
Supplement: Supplementary file 1 — Supplementary note containing the genetic and clinical information for the individual with the FOXP1 duplication. (DOCX 107 kb) [file 13229_2017_172_MOESM1_ESM.docx]

**Supplementary Note**

A duplication spanning FOXP1 was detected at the Stamford Hospital by array-CGH. The duplication is a *de novo* interstitial duplication of ~8.4 Mb, extending from 3p14.1 to 3p12.3 and including *FOXP1* (individual S6, **Supplementary Figure 1**). To our knowledge, *FOXP1* duplications have not been reported in the literature or in Decipher; the variant was evaluated as of unknown significance. The individual carrying the duplication did not meet criteria for ASD on gold-standard assessments (Autism Diagnostic Observation Schedule, 2^nd^ Edition; Autism Diagnostic Interview-Revised) or on clinical examination and is not further discussed in relation to the ASD phenotype. In terms of intellectual and adaptive functioning, S6 displayed borderline cognitive functioning (Full Scale IQ=72), with significantly better developed nonverbal abilities (Standard Score=81) as compared to verbal abilities (Standard Score=65). This individual’s adaptive functioning was similarly developed with scores on the Vineland Adaptive Behavior Scales, 2^nd^ Edition, ranging from 76 to 82 across domains. For the expressive and receptive language domain, individual S6 displayed scores within the range of individuals with the mutations in the gene, including relatively higher scores on expressive versus receptive language measures (Expressive Vocabulary Test, 2^nd^ Edition Standard Score=78, Peabody Picture Vocabulary Test Standard Score=71) and early language delays (single words at 24 months, phrases at 36 months). When looking at gross motor, fine motor and visual-motor integration, S6 displayed motor deficits (VMI-6 Standard Score<45) and relatively delayed motor milestones (crawling at 12 months, walking at 17 months) that were similar to individuals with *FOXP1* mutations.

*
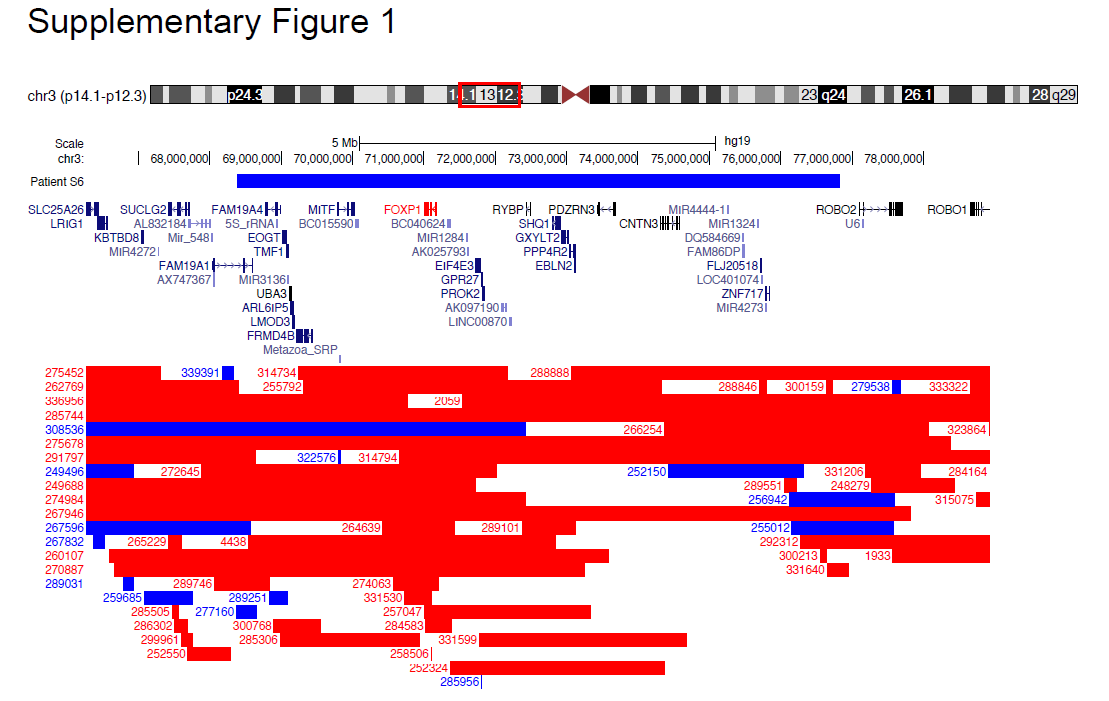
*
