# Supplementary material for: Prospective investigation of FOXP1 syndrome
Source: Mol Autism. 2017 Oct 24;8:57. doi: 10.1186/s13229-017-0172-6 (PMC5655854; doi:10.1186/s13229-017-0172-6)
Supplement: Supplementary file 3 — DSM-5 criteria for ASD in individuals with FOXP1 mutations. (DOCX 18 kb) [file 13229_2017_172_MOESM3_ESM.docx]

**Supplementary Table 2.** DSM-5 Criteria for ASD.

| **DSM 5 – ASD Criteria** | **S1** | **S2** | **S3** | **S4** | **S5** | **W1** | **W2** | **W3** | **W4** | **Total** |
| --- | --- | --- | --- | --- | --- | --- | --- | --- | --- | --- |
| Deficits in Social Communication* | N | N | N | N | N | Y | N | Y | N | 2/9 (22%) |
| Socio-emotional reciprocity | - | + | - | - | - | + | - | + | + | 4/9 (44%) |
| Nonverbal communicative behaviors | - | - | - | - | - | + | - | + | - | 2/9 (22%) |
| Developing and maintaining friendships | + | + | + | + | + | + | - | + | - | 7/9 (78%) |
| Repetitive/Restrictive Behaviors** | Y | Y | Y | Y | Y | Y | N | Y | Y | 8/9 (89%) |
| Stereotypical behaviors | - | - | + | - | - | + | - | + | + | 4/9 (44%) |
| Rigidity/insistence on sameness | - | + | + | + | - | + | - | + | - | 5/9 (56%) |
| Highly restrictive interests/perseveration | + | + | + | + | + | + | - | + | - | 7/9 (78%) |
| Abnormal sensitivities | + | - | + | + | + | + | + | + | + | 8/9 (89%) |
| ASD Diagnosis | N | N | N | N | N | Y | N | Y | N | 2/9 (22%) |

* All three criteria must be met; **Two criteria must be met.
N: Did not meet criteria

Y: Met criteria
